# Achieving Partial Nitritation by Treating Sludge With Free Nitrous Acid: The Potential Role of Quorum Sensing

**DOI:** 10.3389/fmicb.2022.897566

**Published:** 2022-04-27

**Authors:** Cancan Jiang, Xu Wang, Huacai Wang, Shengjun Xu, Wei Zhang, Qingjie Meng, Xuliang Zhuang

**Affiliations:** ^1^Research Center for Eco-Environmental Sciences, Chinese Academy of Sciences, Beijing, China; ^2^College of Resources and Environment, University of Chinese Academy of Sciences, Beijing, China; ^3^The Institute of International Rivers and Eco-Security, Yunnan University, Kunming, China; ^4^Shenzhen Shenshui Water Resources Consulting Co., Ltd., Shenzhen, China; ^5^Institute of Tibetan Plateau Research, Chinese Academy of Sciences, Beijing, China

**Keywords:** low carbon wastewater, partial nitritation, free nitrous acid, acid oxidizing bacteria, quorum sensing

## Abstract

Partial nitritation is increasingly regarded as a promising biological nitrogen removal process owing to lower energy consumption and better nitrogen removal performance compared to the traditional nitrification process, especially for the treatment of low carbon wastewater. Regulating microbial community structure and function in sewage treatment systems, which are mainly determined by quorum sensing (QS), by free nitrous acid (FNA) to establish a partial nitritation process is an efficient and stable method. Plenty of research papers reported that QS systems ubiquitously existed in ammonia oxidizing bacteria (AOB) and nitrite oxidizing bacteria (NOB), and various novel nitrogen removal processes based on partial nitritation were successfully established using FNA. Although the probability that partial nitritation process might be achieved by the regulation of FNA on microbial community structure and function through the QS system was widely recognized and discussed, the potential role of QS in partial nitritation achievement by FNA and the regulation mechanism of FNA on QS system have not been reviewed. This article systematically reviewed the potential role of QS in the establishment of partial nitritation using FNA to regulate activated sludge flora based on the summary and analysis of the published literature for the first time, and future research directions were also proposed.

## Introduction

**Figure 1:**
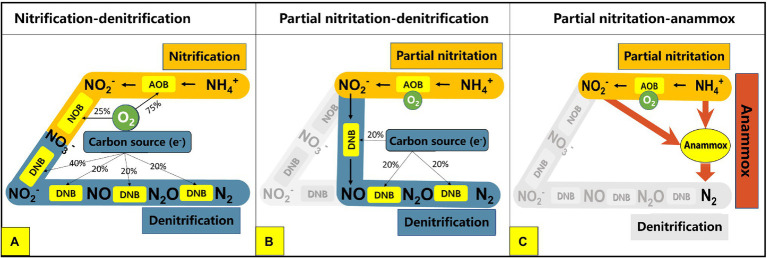
Pathways of different biological nitrogen removal processes: **(A)** traditional nitrification denitrification process; **(B)** partial nitritation denitrification process; **(C)** partial nitritation anammox process. AOB, ammonia oxidizing bacteria; NOB, nitrite oxidizing bacteria; DNB, denitrifying bacteria.

Nitrogen pollution is recognized as a serious environmental problem all over the world. Among the sources of nitrogen pollution in water bodies, the effluent of wastewater treatment plants (WWTPs) has always been an easily overlooked but very important source of pollution (Sun et al., 2016; Zhang et al., 2016). The lower carbon–nitrogen ratio in the influent of WWTP is one of the main reasons for the high concentration of total nitrogen in the effluent (Sinha and Annachhatre, 2006; Hao et al., 2015). This is because the removal of nitrogen in most WWPTs at this stage is mainly dependent on the traditional activated sludge process which generally includes two processes: nitrification and denitrification (Figure 1A; Peng and Zhu, 2006). The nitrification process mainly includes two steps: ammonia oxidation and nitrite oxidation. The ammonia oxidation process is completed by ammonia oxidizing bacteria (AOB), which convert ammonia into nitrite under aerobic conditions. Nitrite oxidation is accomplished by nitrite oxidizing bacteria (NOB), which oxidize nitrite to nitrate. The successful progress of these two steps requires the electron acceptor (O_2_) to be provided by a large amount of aeration. In the denitrification process, denitrifying bacteria (DNB) use carbon sources in the sewage as electron donors to reduce nitrogen from nitrate to nitrite and then further reduce it to nitrogen gas (N_2_) to achieve final nitrogen removal (Figure 1A; Sun et al., 2021). Under such process conditions, an insufficient carbon source in the influent will lead to insufficient denitrification, resulting in an excessively high concentration of total nitrogen (nitrate nitrogen) in the effluent (Rahimi et al., 2020). In order to solve the problems of high energy consumption, carbon source shortage, and low nitrogen removal efficiency faced by traditional nitrogen removal processes, novel processes based on partial nitritation have gradually developed in recent years, such as the partial nitritation and denitrification process (PN/D) and the partial nitritation and anaerobic ammonia oxidation process (PN/A; Duan et al., 2020; Li et al., 2020, 2021; Qiu et al., 2020; Yao et al., 2021). Different from the traditional nitrification and denitrification process, nitrogen removal processes based on partial nitritation only oxidizes ammonia to nitrite, and then directly enters the subsequent nitrogen removal stage, which greatly saves oxygen consumption and carbon source demand, while reducing excess sludge production and greenhouse gas emissions (Sinha and Annachhatre, 2006). For example, compared to the traditional nitrification and denitrification process, the PN/D process has the following advantages: (1) saving 20% oxygen consumption in the nitrification stage; (2) saving 40% carbon demand in the denitrification stage; (3) reducing carbon dioxide production by 20%; (4) reducing excess sludge production by 35 and 55% in the nitrification stage and denitrification stage, respectively (Figure 1B; Jiang et al., 2019). When partial nitritation and anaerobic ammonia oxidation processes are combined, i.e., PN/A process, NH_4_^+^ and NO_2_^−^ produced by partial nitritation can be converted into N_2_ by autotrophic anammox bacteria. Therefore, compared to traditional nitrification and denitrification, PN/A can save 25% oxygen consumption and 100% carbon source demand, greatly reduce process energy consumption and excess sludge production, and at the same time improve the denitrification efficiency of wastewater (Figure 1C; Kang et al., 2020; Adams et al., 2022). At present, many novel nitrogen removal processes based on partial nitritation have been successfully established and applied in engineering, such as SHARON process and CANON process (Ye et al., 2020; Xu et al., 2021).

Stable accumulation of nitrite is the prerequisite and necessary condition for the successful establishment of a novel nitrogen removal process based on PN, which is achieved mainly by suppressing NOB while retaining AOB (Jiang et al., 2018). A variety of regulation methods have been reported to achieve effective NOB inhibition, such as low dissolved oxygen, intermittent aeration, shortening sludge residence time, inhibitor addition, etc. (Liu et al., 2017; Yang et al., 2017; Zhang et al., 2018a; Zhou et al., 2018). In recent years, the method of selective inhibition of NOB by free nitrous acid (FNA) to establish PN has attracted a lot of attention and is considered a very promising technology (Kinh et al., 2017; Pedrouso et al., 2017). FNA is an efficient bacterial inhibitor, but the growth of NOB is more sensitive to FNA than the growth of AOB. The most commonly used methods of FNA to establish PN in laboratory research and practical applications are sidestream treatment of activated sludge by FNA and *in-situ* selective inhibition of NOB by FNA (Zhou et al., 2011; Duan et al., 2020; Ren et al., 2021). In the former method, a part of the mainline activated sludge ~30% was transported to a side-stream treatment unit to be exposed to high levels of FNA in order to inactivate the NOB present in the activated sludge. This approach is generally used to establish the PN process during the treatment of municipal wastewater with low ammonia nitrogen concentration (Wang et al., 2014). A previous study found that after activated sludge was treated with 1.2 mg/l FNA for 18 h, the activity of NOB could be completely inhibited, while the activity of AOB remained above 57% (Jiang et al., 2018, 2019). Other researchers have reported similar results, but with differences in FNA concentrations and treatment times due to diversity in sludge properties and microbial community structure and composition (Duan et al., 2020). For example, Wang et al. (2014) reported that AOB activity could still be maintained at approximately 50% after activated sludge was treated with 1.35 mg/l of FNA for 24 h, while NOB completely lost its activity, while treatment conditions that can completely inactivate NOB while retaining 47% of AOB’s activity are: A FNA concentration of 0.25 mg/l and a treatment time of 18 h was proposed (Wang et al., 2019). Taking advantage of the selective inhibition of NOB by FNA, researchers successfully established PN process in various types of reactors. Wang et al. (2021b) achieved PN by treating 22% of the activated sludge in a sequencing batch reactor (SBR) every day with simulated wastewater as influent, and the accumulation rate of nitrite could reach more than 80% in PN stage (Wang et al., 2021b). In our previous work, a stable PN/D process was successfully established in the continuous flow reactor A^2^O by treating 30% of the sludge daily in the side flow FNA treatment unit. The accumulation rate of nitrite nitrogen could be maintained at more than 78% in PN stage, and the removal efficiency of total nitrogen was increased by 20% compared with the control group (Jiang et al., 2019). Some researchers also used this technology to achieve PN/A process, and the accumulation rate of nitrite could be maintained at 60% ~ 80% in PN stage (Wang et al., 2019). In order to achieve green and low-cost acquisition of FNA, researchers have developed a technology to produce FNA using sludge digested liquid rich in high-concentration ammonia, which could reduce 14% of the total cost and did not lead to a higher N_2_O emission by avoiding external chemicals addition in comparison with commercial supply (Law et al., 2015). In the treatment of high-strength ammonia-nitrogen wastewater, the method of *in-situ* selective inhibition of NOB by FNA is commonly used to construct a PN process. When compared to sidestream inhibition, this method saves space and cost as both the sludge treatment with FNA and the nitritation reaction simultaneously take place in a single reactor and therefore a side-stream sludge treatment unit is not required. Moreover, external NO_2_^−^ and acid are also not required to obtain the required FNA concentrations. Ren et al. (2021) achieved a sustained nitrite accumulation rate above 90% in PN stage when treating mature landfill leachate. To sum up, using FNA to selectively inhibit NOB to establish PN process is a green, efficient, low-cost and highly applicable technology. Understanding how FNA regulates specific microorganisms and their metabolism in the process of PN biological nitrogen removal can further develop its potential and maximize its effectiveness. Although there have been a lot of studies on the establishment of PN by FNA and the underlying mechanisms, they have not been systematic reviewed from the perspective of community mechanisms. Many researches showed that the regulation of activated sludge flora by FNA played an important role in establishing PN process (Zhang et al., 2020; Hu et al., 2021; Luo et al., 2021). And meanwhile, the community structure and functions of activated sludge flora were under the regulation of quorum sensing (QS) and quorum quenching (QQ) which were the molecular communication systems employed by bacteria to regulate their density and organize their collective behavior. Thus, this paper systematically reviewed the function of FNA and QS/QQ systems on PN establishment based on the summary and analysis of the published literatures, and then the potential role of QS/QQ systems in the establishment of PN using FNA to regulate activated sludge flora were discussed, and future research directions were also proposed.

## Function of FNA on PN Establishment: Bactericidal Effect at Single-Bacteria Level

### Bactericidal Mechanisms of FNA

Research on bactericidal effects of FNA on microorganisms at single-bacteria level mainly focuses on its impacts on bacterial metabolism, transmembrane transport of substrates, oxygen acquisition, and oxidative phosphorylation (Laloo et al., 2018; Zheng et al., 2021). FNA can act as a proton uncoupling agent, which is a reagent that stimulates electron transfer, ATP hydrolysis, and inhibits ATP synthesis, ATPase catalysis, and other exchange reactions (Almeida et al., 1995). As a proton carrier, FNA can increase the ability of protons to penetrate cells. Therefore, to maintain the balance of intracellular and intracellular potentials, cells need to expel excess free protons from the intracellular to the extracellular. This process will consume additional ATP, resulting in insufficient ATP to maintain normal metabolism of cells and inhibit cell survival (Anthonisen et al., 1976; Sijbesma et al., 1996; Gao et al., 2016). However, Vadivelu et al. (2006a) found that FNA does not inhibit the ATP production and metabolism of all microorganisms, and the uncoupling effect of FNA alone may not be the only mechanism for FNA inhibition (Vadivelu et al., 2006a,b). In addition, another major action pathway of FNA is to inhibit the growth of bacterial cells by affecting various enzymes in bacterial metabolism. These mainly include enzymes related to nitrogen form conversion, transcription and translation, and decomposition and anabolism (Fang, 1997).

### Differential Impacts of FNA on AOB and NOB

Previous reports suggested that the difference in the inhibitory effect of FNA on AOB and NOB activity may be due to their different regulation ability on the above-mentioned pathways. In terms of the removal of the toxic substrate (FNA), both AOB and NOB have nitrite reductase (*nirK*), a nitrite detoxification gene, which can convert nitrite into non-toxic nitric oxide (NO). However, NOB has two additional nitrite scavenging pathways than AOB, including the nitrite reductase (*nirB/D*) pathway that can convert nitrite into ammonia and nitrite oxidoreductase (*norA/B*) pathway that can convert nitrite into nitrate (Cantera and Stein, 2007). The paradox that AOB has fewer detoxification pathways but higher FNA tolerance has not been definitively concluded in previous studies. Some researchers have explained the mechanism of AOB tolerance to FNA by means of metagenomic and proteomic methods. When *N. eutropha* is treated with FNA, in order to maintain the homeostasis in the bacteria, oxidative stress repair enzymes, denitrification-related enzymes, DNA and protein repair enzymes were up-regulated, and energy production and ion transmembrane transport were strengthened. In addition, β-lactam hydrolase-like proteins and alginate transporters associated with biofilm formation increased in AOB after FNA exposure, suggesting that FNA may promote AOB biofilm formation. However, due to the low detection of NOB-related proteins in the experiment, the molecular biological response mechanism of NOB after FNA treatment has not been fully explained, and the comparison of the regulatory effects of FNA on the same or similar metabolic pathways in AOB and NOB cannot be fully explained (Laloo et al., 2018). Moreover, many studies have reported that under FNA treatment, although the activity of NOB in activated sludge was almost completely inhibited, the survival of NOB was still detected. After applying FNA over an extended period of time the activity and abundance of NOB gradually increased because of NOB adaptation and community shift, resulting in the destruction of PN (Wang et al., 2021a). This indicates the inhibitory effect of FNA on NOB activity is reversible at the community level after prolonged application. Therefore, it might be that FNA affects the structure of the bacterial community by affecting the interaction between AOB, NOB, and the entire activated sludge flora, thus forming a structure of the bacterial community conducive to the growth and reproduction of AOB and at the same time greatly inhibiting the activity of NOB, thus establishing PN.

## Function of QS/QQ Systems During PN Establishment

### QS Systems in PN Process

**Figure 2:**
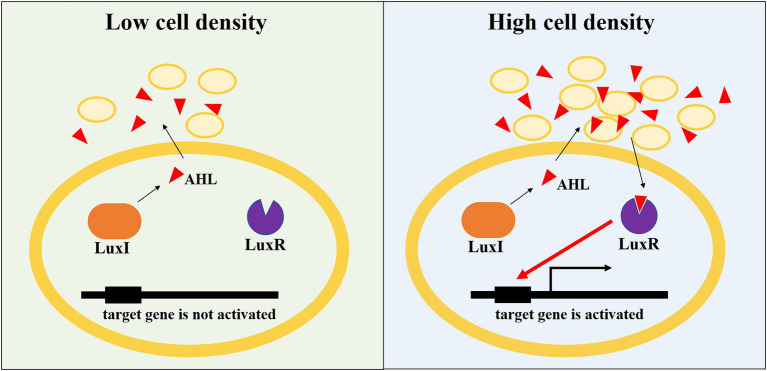
Schematic of AHL-mediated QS in gene express regulation.

As single-celled organisms, the interaction between microorganisms is mainly achieved through quorum sensing (QS; Shrout and Nerenberg, 2012). QS is one of the most important ways of communication between microorganisms in the colony gathering area, and signal molecules are the main molecular substances to realize QS regulation (Huang et al., 2016). Microorganisms synthesize and secrete signal molecules with specific chemical structures through QS system. With the growth and reproduction of microorganisms, the concentration of signal molecules increases continuously. When the density of microorganisms and the concentration of signal molecules reach a certain threshold, the transcription expression of downstream target genes will be regulated. By sensing the expression and secretion of signal molecules, QS system coordinates the physiological behavior and regulates the ecological relationship of the flora, and ultimately determines the bacterial community structure, thus showing the physiological functions and regulatory mechanisms that a single individual bacterium cannot achieve, such as bioluminescence, antibiotic synthesis, biofilm formation, and pathogenic toxicity (Hu et al., 2021). N-acyl homoserine lactones (AHL), oligopeptides, furanborate diesters, quinolones, and γ-butyrolactones are the most common and consistent with the principle of QS signaling molecules in bacteria. AHLs, produced by gram-negative bacteria, are the widely studied signal molecule (Juretschko et al., 2002; Chain et al., 2003; Norton et al., 2008; Gao et al., 2014). It has been reported that more than 100 kinds of *Proteobacteria* can produce AHL signal molecules (Yates et al., 2002). AHL-mediated QS system mainly includes AHL signaling molecules, AHL synthase and AHL receptor. When the concentration of AHLs reaches a certain threshold, it will bind to its receptors and activate the transcription of target genes controlled by the QS system to realize the regulation of QS (Burton et al., 2005; Chen et al., 2018; Maddela et al., 2019). The AHL-mediated QS system is ubiquitous in the nitrification process of activated sludge bacteria (Rice et al., 2016; Shen et al., 2016). AOB and NOB can be regulated by various AHLs with different carbon chain lengths (Table 1). The synthesis and recognition of AHL in nitrifying bacteria are regulated by genes on LuxI/LuxR. LuxI-AHL synthetase catalyzes the condensation reaction between S-adenosylmethionine and acylated acyl carrier protein to generate AHL molecules with homoserine lactone ring as the main body, connecting fatty acyl chains of different lengths and degrees of saturation (Fuqua et al., 2001). When the AHL concentration reaches the threshold concentration, it will combine with the LuxR receptor in the cytoplasm to form the transcriptional regulatory complex LuxR-AHL, and then combine with the specific promoter sequence to regulate the expression of the QS system, thus showing changes in community-level characteristics (Figure 2; Waters and Bassler, 2005; Churchill and Chen, 2011). Recent studies have shown that not only AOB and NOB can produce signal molecules to participate in QS regulation, but different signal molecules can produce different regulatory results when acting on specific nitrifying bacteria (Table 1). Mellbye et al. (2015) reported that the QS regulatory effect of NOB can not only affect the production and consumption of various nitrogen oxides (such as NO, NO_2_, and N_2_O), but also regulate the nitrification completed by AOB (Mellbye et al., 2015, 2016, 2017a,b). Sun et al. (2018) also found that C4-HSL was correlated with the activity of AOB; however, C6-HSL and C8-HSL facilitated the activity of NOB (Sun et al., 2018). A similar result found that C4-HSL promoted nitrite accumulation, while C8-HSL could inhibit AOB activity (Feng et al., 2019). To sum up, these findings indicated that QS played crucial roles in regulating the balance between AOB and NOB, and thus affecting the performance of partial nitritation systems.

**Table 1:** QS systems related to partial nitritation.

Bacteria type	Bacteria species	Signal type	Functions	References
AOB	*Nitrosomonas europaea*	C6-HSL C8-HSL C10-HSL	Resistance against starvation Recovery of starved Nitrosomonas europaea biofilm;	Juretschko et al., 2002; Chain et al., 2003; Burton et al., 2005
	*Nitrosospira multiformis*	C10-HSL C14-HSL 3OC14-HSL	Discovery of a functional AHL synthase in Nitrosospira multiformis	Norton et al., 2008; Gao et al., 2014; Mellbye et al., 2017a,b
	*Nitrosospira briensis*	3-OH-C14-HSL	Unknown	Rice et al., 2016; Mellbye et al., 2017b
	*Nitrococcus mobilis*	Genome contains QS genes and AHLs are not detected	Unknown	Feng et al., 2019
NOB	*Nitrobacter winogradskyi*	C7-HSL C8-HSL C9-HSL, C10-HSL C10:1-HSL	Regulation of possible nitrogen oxide flux and nitrification; Nitrite oxidation, nitrogen oxides metabolism	Mellbye et al., 2016; Shen et al., 2016
	*Nitrobacter vulgaris*	C10:1-HSL	Nitrogen oxides metabolism	Mellbye et al., 2017a,b
	*Nitrospira moscoviensis*	C8-HSL	Unknown	Mellbye et al., 2017b
	*Nitrobacter hamburgensis*	Genome contains QS genes and AHLs are not detected	Unknown	Feng et al., 2019

### QQ Systems in PN Process

**Figure 3:**
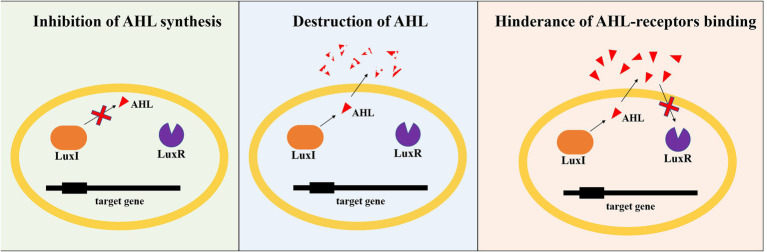
There major types of AHL-mediated quorum quenching.

At bacterial community level, nitrification process is not only regulated by QS, but may also be affected by the interfering or destroying of QS system, i.e., quorum quenching (QQ), which could block the information exchange between cells, and lead to failure of microbial community regulation (Grandclement et al., 2016). The main ways to achieve QQ, as shown in Figure 3, include: (1) inhibiting the synthesis of signal molecules. Blocking the QS system by inhibiting AHL production seems to be a theoretically easy strategy. However, there are just a few experiments targeting the LuxI-type synthase protein that have been published. LuxI-type synthases catalyze the formation of AHL from acyl-ACP and SAM (Geske et al., 2008). Because SAM is an essential and unique step for AHL synthesis, most research on inhibiting AHL synthesis has focused on the use of different analogues of SAM. Thus, damaging AHL synthase and using different intermediate analogues of SAM become the two most commonly reported methods to inhibit AHL synthesis. (2) destructing signal molecules. Chemical, metabolic and enzymatic destruction were the three main types of AHLs destruction (Kalia, 2013). H^+^/OH^−^ were reported to be the primarily chemicals that could destruct AHL structure. There are four main types of AHL degrading enzymes: lactase that breaks the homoserine lactone ring, acylation amide enzyme that cleaves the amide bond and releases fatty acids and homoserine lactones, reductase that converts 3-oxo-AHL to 3-OH-AHL, and cytochrome oxidase that catalyzes acyl chain oxidation. Bacteria are the primary source of the above-mentioned AHL degrading enzymes (Huang et al., 2016; Mellbye et al., 2016). (3) hindering the binding of signal molecules to receptors. The ways of intervening in the binding of signal molecules to receptors mainly include the competition of signal molecule analogs. Those QQ approaches interfere with and disrupt AHL-based QS system, and then inhibit gene expression mediating bacterial desired phenotypes (Chen et al., 2011). Bacteria and chemicals with QQ ability, including *Pseudomonas* sp. 1A1, *Rhodococcus* sp. BH4, paraoxonase, azithromycin, and TiO2, etc., were widely detected in nitrification systems in activated sludge (Kalia, 2013; Kim et al., 2014; Oh and Lee, 2018; Yu et al., 2018). Apparently, FNA can also greatly affect AHL-based QS and QQ systems of microorganisms in activated sludge, and regulate their behaviors and functions at the microbial community level.

## Function of FNA on PN Establishment

**Figure 4:**
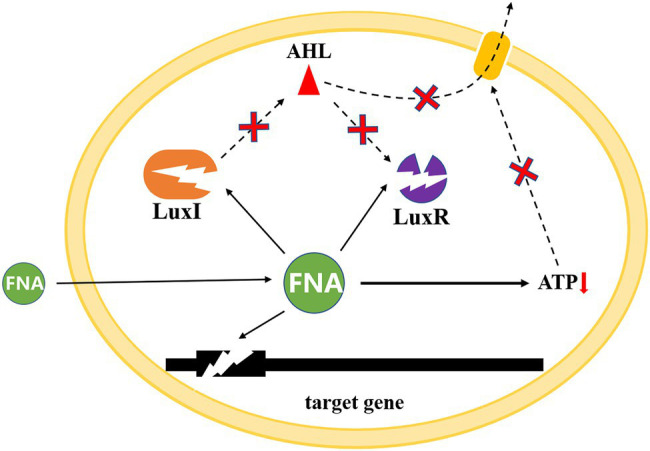
Potential mechanisms of FNA effect on QS system.

Potential QS mechanisms as FNA is able to passively pass through bacterial cytoplasmic membranes and interact with enzymes that contain thiols, heme groups, iron sulfur clusters, phenolic or aromatic amino acid residues, tyrosyl radicals, and amines (Zhou et al., 2008; Gao et al., 2019), the activity of AHL synthase can be easily inhibited, thus influencing the synthesis of AHL. Similar effects might occur on LuxR and receptor protein of AHL, therefore, hinder the regulation of QS on nitrifying bacteria community. Proton permeability of bacterial membranes could increase as FNA diffuse across cell membranes, which might cause the transmembrane electrochemical proton motive force (pmf) to dissipate. The collapse of pmf would limit ATP generation, which further impeded the active transportation of AHL the extracellular and influenced its exudation (Zhou et al., 2007). Moreover, FNA may accumulate inside cells in its anionic form as a result of the near-neutral intracellular pH. This release of protons within the cells would decrease the intracellular pH, which might influence QS and QQ systems during PN establishment (Brul and Coote, 1999; Byers et al., 2002; Vadivelu et al., 2006b). According to Byers et al. (2002), alkaline pH causes the lactone ring to open, resulting in the loss of AHL signal activity. At acidic pH, however, the lactone ring re-cyclizes, and the activity of the AHL signal is reversed. In addition, AHL synthesis has also been discovered to be inhibited by some antibiotics, and nitrite (FNA) has long been known to have antimicrobial properties, which might also contribute to the QQ ability of FNA (Pierson and Smoot, 1982; Rao et al., 2006; Figure 4).

FNA treatment may also result in uneven distribution of signal molecules in extracellular polymeric substances (EPS) in activated sludge (Tan et al., 2014; Ding et al., 2015). EPS is a kind of macromolecular polymer secreted by microorganisms, which is the framework of activated sludge flocculation structure. EPS has the functions of aggregation of microorganisms, enrichment and degradation of organic matter, so it plays a vital role in the distribution of signal molecules in activated sludge (Ding et al., 2015). The structure of EPS from inside to outside is the tightly bound layer (TB), the loose bound layer (LB), and the mucus layer (S), where the tightly bound layer directly contacts the internal microbial cell aggregates. The hierarchical and limited spatial structure of EPS is conducive to the continuous enrichment of signal molecule concentration and reaching the level required for QS regulation (Zhang et al., 2020). Therefore, EPS structure may be the main site of QS regulation in activated sludge. EPS components include proteins, polysaccharides, humus and extracellular DNA, among which proteins and polysaccharides are the main components. Previous studies have shown that the protein and polysaccharide content of LB layer is much higher than that of TB and S layer, but the protein/polysaccharide ratio of TB layer is higher than that of LB layer, indicating that LB layer may be the main area for adsorption of organic matter, but the adsorption of organic matter on TB layer is stronger, and the signal molecules produced by internal bacterial micelles are likely to be firmly adsorbed by TB layer to continuously enrich and reach the QS regulation level (Zhang et al., 2018b; Liu et al., 2021; Zhao et al., 2021; Zheng et al., 2021). Therefore, in order to better explain the QS regulation pathway in activated sludge, it is necessary to further analyze the influence of FNA on the distribution of signal molecules in EPS.

## Conclusion and Prospects

FNA can selectively inhibit NOB, affect the expression of various synthetases in the metabolic processes of AOB and NOB, and cause the up-regulation of the expression of proteins related to the formation of AOB biofilm. The formation and structure of microbial biofilm and EPS are closely related to the synthesis and expression of QS signaling molecules. The regulatory effect of FNA on the structure and function of activated sludge flora is likely to be achieved by regulating the metabolic process directly related to QS in the system, including regulating the synthesis of QS signaling molecules. The spatial distribution of QS signal molecules in activated sludge bacterial micelles was further affected. Regulation of FNA in AOB and NOB can cause secondary regulation of QS in the system. The biological functions of QS signaling molecule synthase directly or indirectly related to QS, EPS synthesis, biofilm formation, nutrient transport and absorption, and nitrogen transformation are further cascade regulated.

Therefore, it is necessary to further reveal the microbial ecology and molecular biology mechanism of FNA that regulates QS in the partial nitritation process from two aspects: (1) changes in the structure and function of the microbial community, the metabolic network relationship, the molecular types of QS signals; (2) the regulation of FNA on physiological functions of AOB and NOB, molecular synthesis of QS signaling, and related metabolic pathways at the cellular and molecular levels. The revelations of these questions will enhance the understanding of FNA regulating the partial nitritation of activated sludge, and provide theoretical guidance for the future application of FNA technology in practice, and lay the foundation for further study of activated sludge ecological system at the molecular level.

## Author Contributions

CJ conceived and wrote the manuscript. XW and HW refined the manuscript. SX, WZ, and QM reviewed the manuscript. XZ supervised the project. All authors have read the final manuscript and approved it.

## Funding

This research was supported by the National Natural Science Foundation of China (Nos. 42177099, 91951108, and 21976197), the Knowledge Innovation Program of Shenzhen (JSGG20191129112812329), and the CAS International Partnership Program (No. 121311KYSB20200017).

## Conflict of Interest

WZ and QM are employed by Shenzhen Shenshui Water Resources Consulting Co., Ltd.

The remaining authors declare that the research was conducted in the absence of any commercial or financial relationships that could be construed as a potential conflict of interest.

## Publisher’s Note

All claims expressed in this article are solely those of the authors and do not necessarily represent those of their affiliated organizations, or those of the publisher, the editors and the reviewers. Any product that may be evaluated in this article, or claim that may be made by its manufacturer, is not guaranteed or endorsed by the publisher.
